# CAR-T cells in solid tumors: engineering, biomarkers, translational pathways and the road ahead

**DOI:** 10.3389/fimmu.2026.1796675

**Published:** 2026-04-01

**Authors:** Nada Saed Homod Al Shaer, Ibraheem Masoud, Aya Tleyjeh, Attas Alawi Al-Attas, Maryam Alawi Al-Attas, Mohammed Imran Khan, Ahmed Yaqinuddin

**Affiliations:** 1 College of Medicine, Alfaisal University, Riyadh, Saudi Arabia; 2 King Faisal Specialist Hospital and Research Center, Jeddah, Saudi Arabia

**Keywords:** antigen heterogeneity, biomarker-driven design, CAR-T cell therapy, immune escape, metabolic reprogramming, solid tumors, spatial omics, tumor microenvironment

## Abstract

Chimeric antigen receptor (CAR) T-cell therapy has transformed the treatment of hematologic malignancies by enabling antigen-specific tumor targeting and durable clinical responses. However, its translation to solid tumors has been limited by fundamental biological barriers, including antigen heterogeneity, poor tumor infiltration, and profound immunosuppressive and metabolic constraints within the tumor microenvironment. These factors collectively drive CAR-T cell dysfunction, exhaustion, and limited persistence, resulting in modest and inconsistent clinical efficacy. This review provides a concept-driven synthesis of recent advances in CAR-T cell therapy for solid tumors, with a specific focus on systems-level engineering strategies that integrate tumor biology, spatial context, and cellular metabolism. We highlight emerging approaches such as *in vivo* CAR programming, logic-gated and multi-antigen receptor designs, and armored CAR-T cells engineered to resist immunosuppression and metabolic stress. Importantly, this review goes beyond descriptive engineering advances by emphasizing the growing role of computational modeling, artificial intelligence, and spatial multi-omics in guiding antigen selection, CAR circuit design, and predictive assessment of therapeutic responses. Unlike prior reviews that primarily summarize antigen targets or CAR engineering strategies, this review integrates biological barriers in solid tumors with emerging engineering solutions to provide a conceptual framework for the development and clinical translation of next-generation CAR-T therapies. By integrating biological determinants of failure with rational engineering solutions, the review delineates translational pathways that link mechanistic insight to clinical implementation. This review advances the field by framing CAR-T therapy for solid tumors as a systems engineering challenge rather than a single-target optimization problem. By integrating immunology, bioengineering, computational sciences, and spatial biology, we outline a roadmap for the development of safer, more durable, and context-aware CAR-T therapies. Continued progress will depend on tumor-specific antigen discovery, interdisciplinary collaboration, and scalable manufacturing and regulatory frameworks, collectively enabling the next generation of effective CAR-T therapies for solid tumors.

## 1 Introduction

Chimeric antigen receptor (CAR) T cell therapy represents a major paradigm shift in the field of immunotherapy and targeted cancer treatment. The groundwork for CAR-T treatment was established in the 1980s when researchers-initiated investigations into modifying immune cells to target cancer; however, these efforts achieved limited clinical success (1). In the early 2000s, researchers accomplished a significant advancement by creating CARs, which entail the genetic alteration of a patient’s T cells to incorporate a chimeric antigen receptor (CAR), thereby enabling these cells to accurately identify and target cancer cells (2).

The clinical impact of CAR-T therapy was first realized in 2017 with the FDA approval of tisagenlecleucel (Kymriah) for relapsed or refractory acute lymphoblastic leukemia (3). This was followed by the FDA approval for three additional CD19-specific CAR T cells: axicabtagene ciloleucel (Yescarta), brexucabtagene autoleucel (Tecartus), and lisocabtagene maraleucel (Breyanzi) for the treatment of various B-cell cancers (4–6). More recently, two BCMA-specific CAR T cell treatments, idecabtagene vicleucel (Abecma) and ciltacabtagene autoleucel (Carvykti), received approval in April 2021 and February 2022 for the treatment of multiple myeloma (7), further cementing the success of CAR-T therapy in hematologic malignancies.

However, despite the major success in treating hematological cancers, targeting solid tumors with CAR-T has faced several obstacles. A major barrier lies in the lack of truly tumor-specific antigens in solid tumors, leading to reduced therapeutic efficacy of CAR-T therapy (8). Additionally, the tumor microenvironment interferes with and suppresses CAR-T cell function, making it difficult for these effector cells to identify and eliminate tumor cells (9). Moreover, CAR-T cells in solid tumors exhibit functional exhaustion, reduced proliferation, and impaired metabolic fitness, resulting in shorter persistence and diminished anti-tumor activity compared with hematologic malignancies (8). As a result, the clinical therapeutic efficacy of CAR-T cells in treating solid tumors remains modest compared to hematological malignancies (10).

Given the rapid expansion of CAR-T research and the growing number of clinical trials in solid tumors, a comprehensive and concept-driven synthesis is needed. In this review, we examine the key biological and translational barriers that limit CAR-T efficacy in solid tumors, map emerging engineering strategies designed to overcome these challenges, and discuss translational pathways and future directions that may enable durable and safe CAR-T therapies for solid malignancies. Importantly, this review is differentiated from recent solid-tumor CAR-T reviews by its organizing framework. Rather than presenting engineering advances as a descriptive list, we integrate biological barriers in solid tumors with emerging engineering strategies and biomarker-guided translational approaches. This systems-level perspective aims to provide a conceptual roadmap for the development and clinical translation of next-generation CAR-T therapies for solid tumors.

## 2 Determinants of CAR-T failure in solid tumors: four biological pillars

In contrast to hematologic malignancies, solid tumors present several biological and clinical barriers that severely limit the efficacy of CAR T-cell therapy. Clinical efficacy of CAR T-cell therapy in solid tumors is limited by antigen heterogeneity, impaired tumor penetration, physical stromal barriers, and immunosuppressive cues within the tumor microenvironment (11–13).

### 2.1 Antigen heterogeneity and immune escape

A major limitation of CAR-T cell therapy is the development of tumor resistance against constructs that are designed to target a single antigen. Initially, those specific CAR-T cells can result in high response rates. However, a significant proportion of patients experience relapse because malignant cells eventually lose part of or all of the target surface marker, which is a phenomenon known as antigen escape (14).

One important contributor to the issue is the heterogeneous expression of tumor antigens in solid tumors, which creates risks for on-target, off-tumor toxicity (15–17). In B-cell malignancies and multiple myeloma, B-cells and plasma cells have high specific expression of CD19 and BCMA, which makes targeted CAR-T cells effective. However, CAR-T cells eradicate any cell displaying these antigens on its surface, whether the cell is normal or cancerous (16). This off-tumor activity can result in B-cell or plasma cell aplasia, which is generally tolerated because the therapeutic benefit outweighs the adverse effects. In contrast, solid tumor antigens are often expressed on healthy cells in different tissues, meaning that CAR-T cell activity against non-malignant cells can lead to intolerable, potentially fatal toxicities, and that’s why their use against solid tumors is limited (15, 16). Consistent with this, CAR-T-associated toxicities can affect multiple organ systems and frequently arise from on-target, off-tumor antigen recognition. For example, CAIX-targeted CAR-T therapy resulted in hepatic toxicity due to recognition of CAIX expressed at low levels on normal bile duct epithelium, with liver biopsies confirming CAR-T infiltration and inflammation surrounding bile ducts (18, 19).

In addition to that, the selective pressure imposed by spatial and temporal antigen heterogeneity in solid tumors, both within individual tumor masses and across primary and metastatic sites, limits the efficacy of single-target CAR-T therapies by enabling the selective survival and expansion of antigen-low or antigen-negative tumor subclones (16, 20, 21). Beyond antigen diversity, heterogeneity in solid tumors also reflects unequal antigen retention across distinct intratumoral regions, a phenomenon termed spatial heterogeneity (22). The heterogeneity is also found in the tumor microenvironment and is defined by a spectrum of neoantigen expression, the differential infiltration of immunosuppressive versus effector cells, the quality of the vascular network, and the specific composition of the metabolic and cytokine milieu. Such immunological heterogeneity could arise from different factors such as genetic instability, epigenetic modification, and exposure to microenvironmental stress (23). Another important factor in the tumor microenvironment is that there is a difference in oxygen levels between the necrotic center of the tumor and the aberrant vasculature around it, which corresponds to metabolic shifts in glucose, lactate, and pH. These gradients drive the tumor cells in different zones to release different chemotactic factors and have different expressions of immunosuppressive mediators. For example, cells that are far from the blood supply attract tumor-associated macrophages by releasing damage-associated molecular patterns (DAMPs) and direct their differentiation within the hypoxic tumor core (24). Furthermore, malignant cells with high glycolytic activity express more immunosuppressive mediators in addition to shifting their metabolism pathway to anabolic reactions (23).

As a result of these spatial barriers, following local administration, CAR-T cells often become sequestered at the tumor periphery, and only a small fraction reach the tumor core (25, 26). Ultimately, overcoming the spatial heterogeneity of both antigen retention and TME states remains a critical challenge for the development of resilient, next-generation CAR-T therapies that are capable of navigating geographical zones with variable antigen density and metabolic hostility.

Antigen escape further limits CAR-T cell therapy, as CAR-T cell activation and cytotoxicity are highly dependent on target antigen density, with reduced antigen expression significantly compromising therapeutic efficacy. This limitation is further exacerbated by the inferior sensitivity of CARs compared with native T-cell receptors at low antigen densities, enabling tumor cells expressing subthreshold levels of target antigen to evade immune recognition and drive resistance and relapse (11, 27). There are several mechanisms of escape, ranging from the selection of pre-existing target-negative clones and antigen gene mutations to alternative splicing, epitope masking, and defects in antigen processing (28).

Among these, one particularly complex mechanism is trogocytosis, a biological process involving the intercellular transfer of plasma membrane fragments. Through this interaction, CAR-T cells strip antigens from the tumor surface, which reduces the antigen density on the tumor and leads to resistance. At the same time, the CAR-T cells acquire the tumor antigen, which can trigger self-activation, functional exhaustion, and even fratricide, where CAR-T cells target and kill one another (28).

### 2.2 Impaired trafficking and physical exclusion

A primary physical constraint is the development of a dense fibrogenic TME by stromal cells, specifically cancer-associated fibroblasts (CAFs) (29). Upon activation by transforming growth factor β (TGF-β), these cells stimulate the production of extracellular matrix (ECM) proteins, which restrict T-cell motility and trafficking (30). The ECM consists of stromal cells, fibrous proteins, glycoproteins, proteoglycans, and polysaccharides, providing structural support to the tumor and separating it from surrounding tissues (31, 32).

The effective localization and migration of T-cells is a fundamental prerequisite for antitumor immune surveillance (33). However, unlike hematological malignancies, the success of CAR-T cell therapy in the solid tumor setting is severely restricted by the inability of engineered cells to traffic to and infiltrate the neoplastic mass. This failure is driven by a combination of the immunosuppressive tumor microenvironment, which limits CAR-T cell penetration and mobility (14). Specifically, their entry is obstructed by a lack of optimal inflammatory and chemokine signals, the presence of an abnormal vasculature, and the physical exclusion imposed by a dense stromal matrix surrounding the tumor cells (34).Consequently, the exclusion of CAR-T cells results from a coordinated series of defects in T-cell trafficking and extravasation (35). More recently, single-cell transcriptomic analyses have elucidated the mechanisms underlying the limited infiltration of CAR-T cells into solid tumors, revealing that immune-excluded tumor regions exhibit transcriptional enrichment in activated fibroblast programs, suppressive myeloid cell states, and aberrant chemokine signaling, which collectively impede T-cell trafficking and retention (36, 37). Concomitantly, single-cell profiling of tumor-infiltrating lymphocytes discloses a depletion of effector-like T-cell states alongside enrichment in dysfunctional or exhausted phenotypes, driven by chronic antigen exposure, hypoxia, and metabolic stress, all of which constrain durable CAR-T cell functionality within the tumor microenvironment (38, 39). Collectively, these observations demonstrate that suboptimal CAR-T cell infiltration arises not only from physical impediments but also from pre-existing cellular ecosystems that actively restrict T-cell trafficking and functionality (36).

Spatial omics technologies now enable the precise mapping of the localization of these cellular subsets within the solid tumor microenvironment. Spatial transcriptomics and multiplexed imaging technologies have revealed CAR-T cells forming distinct infiltrates that co-localize with antigen-expressing tumor regions, while concurrently delineating the barriers imposed by cancer-associated fibroblasts and suppressive myeloid cells (40).High-resolution spatial profiling of the tumor immune microenvironment reveals that CAR-T cells preferentially localize within niches featuring favorable cytokine gradients and diminished extracellular matrix rigidity, whereas their exclusion from hypoxic regions enriched with immune checkpoints foreshadows therapeutic resistance (41). Collectively, single-cell and spatial omics datasets furnish a strategic framework for engineering CAR-T cells and modulating the tumor microenvironment to enhance infiltration, persistence, and therapeutic efficacy against solid tumors.

Within solid tumors, dysregulated angiogenic signaling generates a vasculature that is structurally aberrant and functionally inefficient. These vessels are characteristically tortuous, dilated, and hyperpermeable, resulting in chaotic blood flow and elevated interstitial fluid pressure. Such conditions impede the transvascular migration of immune cells by creating a physical barrier to extravasation and reducing the expression of essential endothelial adhesion molecules, and this blockade significantly limits the efficacy of immunotherapeutic interventions, including CAR-T cell therapies (42). Furthermore, this aberrant vascular architecture frequently creates regions of hypoxia and nutrient deprivation, which further inhibit immune cell trafficking (43).

In addition to physical vascular barriers, solid tumors actively manipulate chemokine signaling to establish an immunosuppressive landscape that excludes CAR-T cells (44). While chemokines are key mediators of T-cell homing, tumors frequently downregulate the expression of ligands required for T-cell recruitment, such as CXCL9 and CXCL10, while simultaneously upregulating signals that preferentially attract immunosuppressive populations like Tregs and MDSCs (45). For instance, endothelial cells within the TME often overexpress CCL22, a chemokine that recruits Tregs rather than effector T-cells (46). This creates a functional “mismatch” between the chemokine receptors expressed on CAR-T cells and the ligands secreted by the tumor, leading to poor migration into the TME (47, 48). Additionally, the upregulation of immune checkpoint molecules, such as programmed death-ligand 1 (PD-L1), further impairs CAR-T cell function upon arrival (49, 50).

### 2.3 Immunosuppressive tumor microenvironment

The tumor microenvironment (TME) represents one of the most significant barriers to CAR-T cell therapy efficacy because it hinders CAR-T cell trafficking to the target site, disrupts metabolic function, and creates an immunosuppressive environment that promotes T-cell exhaustion (51). Previous studies have indicated that merely 1-2% of CAR-T cells successfully infiltrate the tumor core, leading to a significant reduction in killing efficiency (52). The resistance mechanisms within the TME can be categorized into three distinct layers: physical exclusion, cellular immunosuppression, and metabolic hostility.

Within this cellular layer, the TME comprises tumor and stromal cells together with immunosuppressive myeloid and lymphoid populations (53). Concurrently, cancer and immune cells release secreted factors, including cytokines and chemokines, into the TME (54). These factors influence the TME by regulating immune cell trafficking, polarization, activation, cell growth, and the overall inflammatory state (55).

MDSCs are immature myeloid cells that exist as either mononuclear (M-MDSCs) or polymorphonuclear (PMN-MDSCs) subsets. M-MDSCs share similarities with TAMs, while PMN-MDSCs phenotypically resemble neutrophils (56). MDSCs impair T-cell activity through high PD-L1 expression, secretion of suppressive cytokines, and sequestration of essential nutrients (56). A key metabolic mechanism involves the accumulation of cysteine, which is required for T-cell activation. The resulting depletion of cystine in the TME caused by MDSC sequestration prevents the activation of both native T cells and CAR-T cells (56, 57). Clinical evidence shows that lower MDSC levels in the tumor correlate with improved CAR-T treatment outcomes, highlighting the inhibitory role MDSCs play in the TME (58).

Regulatory T cells (Tregs), characterized by the expression of CD4, CD25, and FoxP3 (59), play a vital physiological role in maintaining immune homeostasis and influencing peripheral tolerance to prevent autoimmunity (60, 61). However, in the context of cancer, their role is complex. Treg populations often increase during tumor progression, a finding correlated with poor prognosis in several solid tumors (62, 63). While Tregs may help prevent chronic inflammation, which is a state that can induce mutations and tumor progression (64), they interfere with cytotoxic T-cell–mediated clearance of cancer cells and secrete suppressive cytokines (65).

The enzyme IDO catalyzes the conversion of tryptophan into kynurenine; this metabolic alteration suppresses effector T and natural killer (NK) cells while recruiting and activating immunosuppressive MDSCs (66). Additionally, the interactions between tumor cells and the immune system are complex, with evidence suggesting that tumor cells induce CAR-T cell dysfunction through the secretion of immunosuppressive extracellular vesicles (67).

### 2.4 T-cell exhaustion and metabolic stress

The metabolic deregulation within solid tumors imposes severe physiological stress on infiltrating T-cells. Rapidly dividing tumor cells deplete the majority of available glucose and oxygen, creating a hypoxic and nutrient-deficient environment (68).

Hypoxia is prevalent in the TME, affecting the function of all resident cells (69). Similar to nutrient deprivation, hypoxia reduces the activation of effector cells, such as CD8+ T cells and NK cells, potentially causing cell death and diminished cytokine production (70, 71). Furthermore, low oxygen levels favor suppressive immune populations, including Tregs and M2 macrophages (53, 72).

The Warburg effect also impacts TME acidity, as the excessive production of lactic acid via glycolysis lowers the pH (73).Elevated lactate levels produced by these hypermetabolic tumor cells have been associated with the dampening of T-cell signaling mediated by the nuclear factor of activated T cells (NFAT) (74, 75) and the expansion of Treg cells (76, 77). High lactate concentrations correlate with decreased cytokine production in T and NK cells. Conversely, lactate dehydrogenase interacts with FoxP3, providing Tregs with a metabolic advantage in the TME (53). Ultimately, these metabolic pressures decrease CAR-T cell therapy efficacy, overriding the antitumor response (78–80).

Even following successful tumor infiltration, therapeutic efficacy is often compromised by T-cell exhaustion (81, 82). Clinical observations consistently demonstrate that patients with solid tumors exhibit significantly reduced CAR-T cell expansion and shorter persistence compared to those treated for hematological malignancies (83). It is characterized not only by the upregulation of immune inhibitory receptors but also by weakened effector functions, diminished self-renewal capacity, and profound alterations in epigenetics, transcriptional programming, and metabolism (84).

The solid tumor environment presents a unique challenge due to its high density of cancer cells, which creates an environment of intense and continuous antigen stimulation (85, 86). While the precise mechanisms linking repeated stimulation to anergy remain under investigation, recent evidence suggests that the inhibition of mitochondrial oxidative phosphorylation plays a critical role, leading to epigenetic remodeling and the downregulation of effector genes (87). These metabolic disruptions, particularly mitochondrial depolarization, drive T-cells toward a state of terminal exhaustion (86). This receptor accelerates exhaustion not only through direct interaction with PD-L1 on tumor cells, which can trigger apoptosis (88–91).

T-cell exhaustion is driven by a coordinated transcriptional program that emerges during chronic antigen stimulation. Rather than representing a single signaling pathway, exhaustion reflects a hierarchical regulatory network that progressively reshapes gene expression, epigenetic accessibility, and cellular metabolism (92). A central driver of this state is the NFAT family; under conditions of chronic stimulation, NFAT shifts from forming cooperative heterodimers with AP-1 to forming homodimers, which directly promote the expression of inhibitory receptors such as PD-1, LAG-3, and TIM-3 (93–95). This dysfunctional program is further reinforced by downstream transcription factors, including IRF4, BATF, and NR4A1 (96–98). The balance between T-bet and EOMES also plays a critical role; while T-bet represses PD-1 in functional effector cells (99), exhausted cells typically downregulate T-bet and upregulate EOMES, with a high EOMES: T-bet ratio serving as a hallmark of the exhausted phenotype (100, 101).

Furthermore, TCF-1 is essential for maintaining a pool of stem-like, pre-exhausted progenitors that sustain the response (102, 103), whereas the master regulator TOX fixes cells in a permanent state of exhaustion through extensive chromatin remodeling and the upregulation of inhibitory markers (104–109). Essentially, TOX expression is induced by NFAT, thereby linking chronic stimulation directly to the epigenetic enforcement of dysfunction. Once established, TOX drives the transcription of a distinct exhaustion program, promoting the expression of inhibitory receptors (TIM-3, LAG-3) alongside critical transcription factors such as EOMES, TCF-1, and CD38 (92).

**Figure 1:**
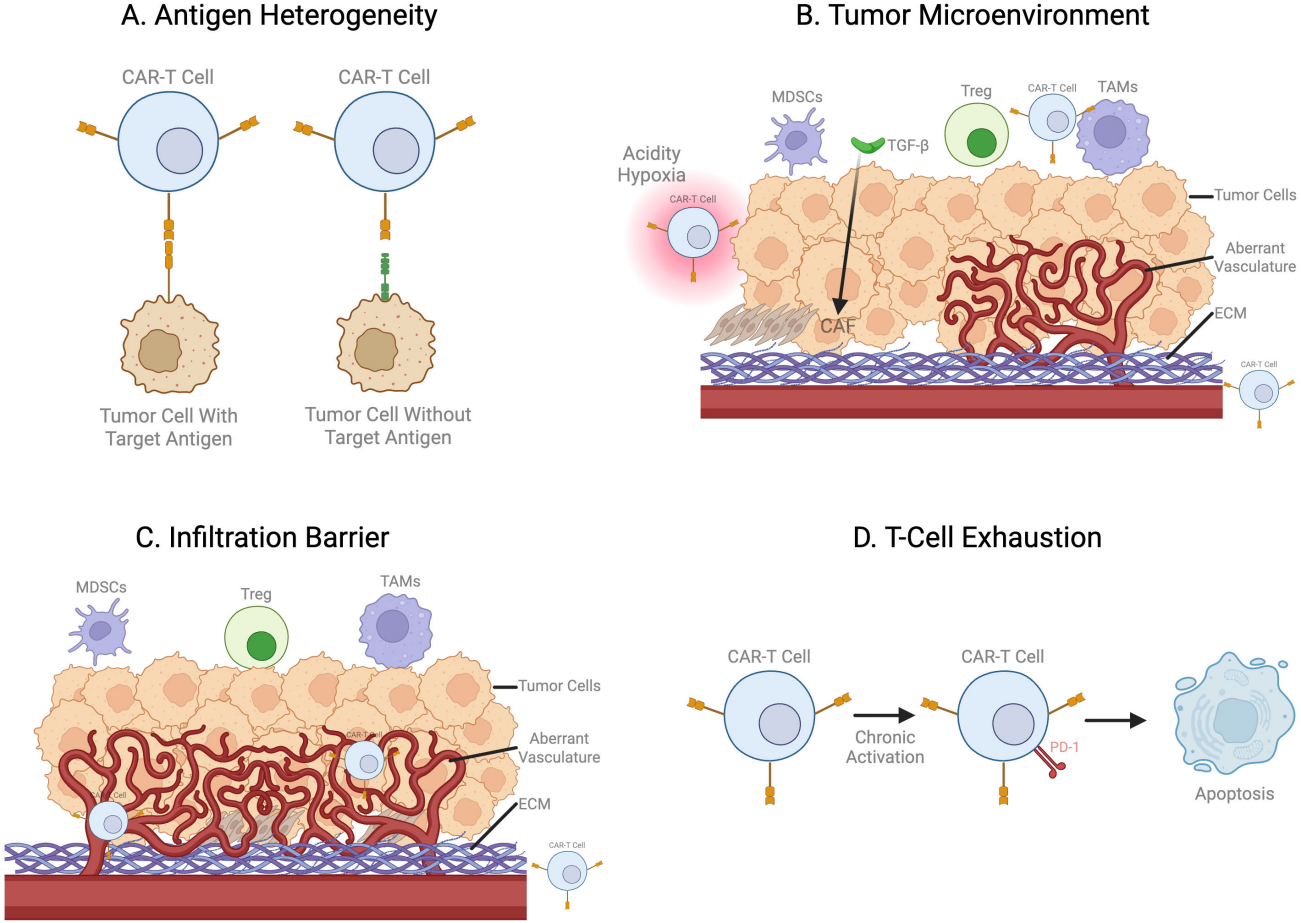
Schematic representation of key barriers in solid tumor CAR-T cell therapy: **(A)** antigen heterogeneity, **(B)** immunosuppressive tumor microenvironment, **(C)** obstacle to trafficking and infiltration, and **(D)** T-cell exhaustion. Created with BioRender.com.

Together, these transcriptional networks link chronic antigen exposure to stable epigenetic and metabolic programs that enforce CAR-T cell dysfunction within the solid tumor microenvironment. Collectively, these biological barriers highlight the need for next-generation engineering strategies capable of overcoming these limitations, as discussed in the following sections. The major biological barriers contributing to CAR-T dysfunction in solid tumors are summarized schematically in **Figure 1**, while a structured overview of the key biological and translational barriers and representative strategies to address them is provided in **Table 1**.

**Table 1:** Major translational barriers limiting CAR-T therapy in solid tumors.

Translational barrier	Biological basis	Clinical consequences	Potential solutions	Key references
Antigen Heterogeneity and Immune Escape	Solid tumor antigens are heterogeneously expressed across tumor cells and between primary/metastatic sites (spatial and temporal heterogeneity). Antigen-negative or antigen-low subclones survive and expand under selective pressure. Mechanisms include antigen gene mutations, alternative splicing, epitope masking, trogocytosis, and defects in antigen processing.	Initial high response rates followed by relapse due to antigen escape. CAR-T cells with insufficient sensitivity at low antigen densities fail to eliminate residual tumor. On-target, off-tumor toxicity in normal tissues expressing the same antigen (e.g., hepatic toxicity with CAIX-targeted therapy).	Logic-gated CAR designs (AND/OR/IF-THEN gates), multi-antigen targeting, SynNotch circuits, AI/ML-guided multi-antigen combination selection, CAR affinity tuning.	(11, 15, 16, 20, 28, 115, 117)
Impaired T-cell Trafficking and Physical Exclusion	Dense fibrogenic ECM produced by cancer-associated fibroblasts (CAFs) activated by TGF-β restricts T-cell motility. Aberrant tumor vasculature (tortuous, hyperpermeable, elevated interstitial fluid pressure) impedes transendothelial migration. Chemokine mismatch: tumors downregulate CXCL9/CXCL10 and upregulate CCL22, attracting Tregs over effector T cells.	Limited CAR-T cell homing and infiltration into solid tumors leads to insufficient tumor engagement, reduced cytotoxic activity, and diminished therapeutic efficacy, with CAR-T cells often remaining at the tumor periphery or within permissive niches rather than penetrating immune-excluded regions.	Engineering CAR-T cells with chemokine receptor matching (e.g., CXCR2/CXCR3 expression), stromal-targeting strategies (e.g., FAP-directed approaches), ECM-modifying enzymes (e.g., heparanase), vascular normalization therapies, and regional or intratumoral CAR-T delivery to enhance trafficking and tumor infiltration.	(30, 45, 46, 52, 118, 121)
Immunosuppressive Tumor Microenvironment (Cellular)	The TME harbors immunosuppressive populations including MDSCs (impairing T-cell activity via PD-L1, suppressive cytokines, cystine sequestration), Tregs (secreting suppressive cytokines, expanding during tumor progression), and tumor-associated macrophages. IDO converts tryptophan to kynurenine, suppressing effector T/NK cells. Tumor cells secrete immunosuppressive extracellular vesicles.	Reduced CAR-T expansion and persistence within the tumor. Failure of cytotoxic response despite successful tumor infiltration. Lower MDSC levels correlate with improved CAR-T outcomes, highlighting their inhibitory role. Poor prognosis in multiple solid tumors correlates with elevated Treg populations.	Armored CAR-T cells secreting IL-12, IL-18, or IL-15 to reshape the tumor milieu. Dominant-negative TGF-β receptors (dnTGFβRII). PD-1–CD28 switch receptors. Combination with checkpoint blockade (PD-1/PD-L1 inhibitors). Oncolytic viruses to reduce immunosuppression.	(56, 58, 65, 66, 122, 198)
Metabolic Hostility and T-cell Exhaustion	Tumor cells deplete glucose, oxygen, and key amino acids (arginine, tryptophan, cystine), creating a hypoxic, nutrient-deprived TME. Warburg-effect lactate accumulation lowers pH, dampens NFAT signaling, and promotes Treg expansion. Chronic antigen stimulation drives exhaustion via NFAT/TOX/NR4A1 transcriptional programs, mitochondrial depolarization, and upregulation of inhibitory receptors (PD-1, LAG-3, TIM-3).	Significantly reduced CAR-T expansion and shorter persistence in solid tumors versus hematologic malignancies. Diminished cytokine production and cytotoxicity. Epigenetic fixation of exhaustion state limits reversibility. High EOMES:T-bet ratio and TOX expression serve as hallmarks of terminal exhaustion.	Metabolic rewiring: overexpression of amino acid transporters (LAT1, xCT), GLUT1, arginine biosynthesis enzymes. ACAT1 inhibition and CD36 deletion to improve lipid metabolism. Engineering resistance to lactate, adenosine, and kynurenine. NR4A- or TOX-knockout approaches. Epigenomic profiling to identify memory-like subsets for manufacturing.	(73, 74, 84, 87, 128, 129, 133)
Manufacturing, Scalability, and Cost	Autologous CAR-T production requires leukapheresis, viral transduction, ex vivo expansion, and extensive quality control. Heavily pretreated solid-tumor patients often have dysfunctional T cells, reducing expansion success and prolonging vein-to-vein time (2–4 weeks). Next-generation armored/multi-antigen constructs require larger vectors and complex engineering, lowering transduction efficiency.	High per-patient costs (often hundreds of thousands of USD) limit access. Prolonged production timelines critically impact treatment initiation. Repeated dosing (up to 18 weekly infusions for CNS tumors) amplifies logistical burden. Specialized centers with intensive monitoring capacity are required, limiting geographic availability.	Allogeneic ‘off-the-shelf’ CAR-T platforms (multiplex CRISPR/base editing to disrupt TCR/HLA). Fully closed and automated manufacturing platforms. *In vivo* CAR-T engineering via nanoparticle delivery. Decentralized point-of-care manufacturing. Internationally harmonized regulatory guidelines.	(48, 157, 162–164, 174)
Delivery Route and Patient Selection	Systemic IV delivery leads to poor trafficking, dilution, and reduced effective dose. The blood-brain barrier and dense ECM in CNS tumors further impede CAR-T distribution. Anatomical constraints of cavitary tumors (peritoneal, pleural, CNS) influence optimal delivery route. Locoregional administration improves tumor exposure but requires interventional procedures.	Inconsistent clinical responses partly attributable to suboptimal delivery. Patients with CNS malignancies require neurosurgical access, restricting eligibility. Intra-arterial delivery for GI malignancies requires specialized procedural expertise. Multi-dose regimens complicate clinical workflow and manufacturing capacity.	Locoregional delivery strategies (intratumoral, intraperitoneal, intrapleural, intracerebroventricular). Intra-arterial delivery for hepatic/GI malignancies. Biomarker-guided patient selection (TME profiling, antigen expression mapping). Route-specific eligibility criteria integrated into trial design.	(119, 120, 138, 161, 167)
Regulatory, Economic, and Regional Barriers	No CAR-T therapy for solid tumors has received marketing approval, reflecting challenges in antigen specificity and TME immunosuppression. Stricter regulatory requirements in the US and Europe slow late-phase translation. The Gulf region faces limited GMP-compliant manufacturing facilities and prolonged vein-to-vein times.	Most global CAR-T solid tumor trials remain in early-phase. High attrition rates and uncertain efficacy, particularly for smaller companies. Hidden costs (facilities, staffing, quality control) impede scaling. Advanced cell-therapy centers concentrated in urban hubs create access inequities, especially in lower-income regions.	Investment in decentralized/point-of-care manufacturing facilities. Standardized automated platforms to reduce costs. Inducible safety mechanisms (suicide switches) to support regulatory progression. Government investment in regional manufacturing (Saudi Arabia, UAE, Qatar). Internationally harmonized regulatory frameworks.	(141, 143, 149, 150, 196)

## 3 Engineering strategies to overcome biological barriers

Although CAR T-cell therapy has transformed the treatment of several hematologic malignancies, its translation to solid tumors remains constrained by the biological barriers outlined in Section 2. These challenges have driven the development of next-generation CAR T-cell strategies focused on improving tumor specificity, functional persistence, metabolic fitness, and manufacturing scalability, increasingly guided by insights from multi-omics and spatial profiling (110).

### 3.1 Logic-gated CAR designs and multi-antigen targeting

To address antigen heterogeneity and reduce immune escape, next-generation CAR T-cell designs increasingly incorporate logic-based antigen sensing, in which T-cell activation depends on defined combinations of antigen inputs (111). CAR signaling has been tuned through modifications in receptor affinity, co-stimulatory architecture, and surface expression to balance tumor sensitivity with safety and functional persistence (112–114). OR-gated CARs recognize either of two TAAs, broadening target coverage and reducing antigen-loss escape; however, their application remains limited by narrow therapeutic windows (115, 116). AND-gated CARs require simultaneous engagement of two antigens co-expressed on the same tumor cell. Activation occurs only when both signals are present, reducing toxicity against single-antigen normal tissues (115). IF-THEN circuits restrict potent activity to the tumor microenvironment. In these systems, recognition of a highly tumor-specific antigen (IF) induces expression of a second CAR (THEN) targeting a more broadly expressed antigen, enabling tumor-restricted activation (112, 115). A leading example is the SynNotch system, where ligand engagement triggers proteolytic cleavage and release of a transcription factor that drives expression of a secondary CAR. SynNotch CARs have shown strong preclinical efficacy in glioblastoma, improving selectivity and overcoming intratumoral heterogeneity (117). However, the clinical translation of these designs may be limited by increased engineering and manufacturing complexity, as multi-component circuits require precise control of receptor expression and function.

### 3.2 Trafficking engineering & delivery strategies

Systemic intravenous delivery often results in poor trafficking and dilution before reaching the tumor, restricting the effective dose range and reducing efficacy. In contrast, locoregional administration has demonstrated superior tumor exposure, enhanced antitumor responses, and a favorable safety profile (118). Cavitary routes, including intraperitoneal, intrapleural, and intracerebroventricular infusion, are advantageous for tumors confined to anatomical spaces and have shown improved persistence and safety (119, 120). Combinatorial strategies are being developed to equip CAR T-cells with chemokine receptors like CCR8 to improve tumor homing (121).

### 3.3 Armored CARs & checkpoint resistance

To counteract the immunosuppressive solid-tumor microenvironment that restricts CAR-T cell activation, persistence, and cytotoxicity, armored CAR-T cells are engineered to deliver additional payloads, such as cytokines or modified receptors, that enhance fitness and resist inhibitory signaling. These modifications can improve CAR-T-cell proliferation, survival, recruitment of endogenous immune cells, and resistance to suppressive cytokines (122, 123). CAR-T cells engineered to secrete IL-12 or IL-18 can reshape the tumor milieu by activating macrophages, NK cells, and endogenous T cells, thereby broadening antitumor immunity beyond the infused product. IL-15-expressing CAR T cells have demonstrated improved persistence and stem-like phenotypes (124). In addition to that, Synthetic-notch circuits enabling tumor-restricted IL-12 or IL-2 production have emerged as promising solutions, delivering potent stimulation only upon tumor antigen engagement (123).

A second major class involves dominant-negative receptors, or switch receptors, that neutralize inhibitory pathways. Examples include PD-1-CD28 switch receptors that convert PD-L1 signaling into a costimulatory cue and dominant-negative TGF-β receptors (dnTGFβRII) that block TGF-β-mediated suppression (125, 126). These modifications have consistently enhanced CAR-T cell expansion and effector function in solid-tumor models. Toxicities from IL-12, IL-15, and dnTGFβRII in early trials highlight the gap between murine and human immunobiology. These findings underscore that strategies designed to enhance potency may also narrow the safety margin, requiring careful control of immune activation in clinical settings. To date, no armored CAR-T product has progressed beyond early-phase clinical testing (123).

### 3.4 Metabolic rewiring to limit CAR-T exhaustion

Metabolic engineering aims to enhance CAR T-cell fitness under hostile conditions imposed by the solid tumor microenvironment (TME), which is characterized by nutrient deprivation, hypoxia, and the accumulation of immunosuppressive metabolites that impair T-cell activation, survival, and cytotoxicity (127, 128).

Tumors deplete key amino acids such as arginine, tryptophan, and cystine, leading to defective proliferation and impaired cytotoxic function. Engineered CAR T cells can be modified to overexpress amino acid transporters such as LAT1 and xCT, improving amino acid uptake even under scarcity (129). Others have been equipped with enzymes such as argininosuccinate synthase or ornithine transcarbamylase to allow internal arginine synthesis, supporting sustained effector function (130).

Enhancing glucose import (e.g., GLUT1 overexpression) or modulating glycolytic regulators can partially overcome this (131). However, excessive glycolysis can drive terminal differentiation, so recent work has focused on balancing glycolytic activity with mitochondrial fitness and glycogen storage to support memory-like CAR-T-cell phenotypes (132).

Beyond amino acids and glucose, lipid metabolism also plays a critical role in shaping CAR-T-cell persistence. Tumor lipids and oxidized fatty acids contribute to T-cell exhaustion and ferroptosis. Engineering strategies such as ACAT1 inhibition, to increase membrane cholesterol, or CD36 deletion, to prevent lipid peroxidation, have enhanced persistence and cytotoxicity in preclinical models (133, 134).

CAR-T cells engineered to degrade or resist inhibitory metabolites such as lactate, adenosine, and kynurenine show improved expansion and tumor control in preclinical models (128). Ongoing work is focused on integrating multiple metabolic modifications to overcome nutrient competition and immunosuppression in the TME (128). Nevertheless, the clinical feasibility of such approaches remains uncertain, as increasing engineering complexity may complicate manufacturing, regulatory evaluation, and safety assessment.

### 3.5 Combination strategies to remodel the tumor microenvironment

Combination therapies are increasingly explored not as additive modalities, but as enabling strategies designed to dismantle extrinsic barriers that constrain CAR-T cell infiltration, function, and persistence in solid tumors. T-cell dysfunction and exhaustion within the hostile and heterogeneous solid tumor microenvironment represent major limitations to CAR T-cell therapy (**Table 2**) (110). Checkpoint blockade strategies, including the combination of CAR T cells with PD-1/PD-L1 inhibitors or dual immune checkpoint blockade, have therefore been explored to partially restore CAR T-cell function. However, early studies indicate that checkpoint inhibition alone is often insufficient to overcome barriers such as poor tumor infiltration and profound immunosuppression, and concerns regarding immune-related toxicities and long-term efficacy remain (135).

**Table 2:** Engineering strategies in development (next-gen CARs) and their status.

Engineering strategy	Subtype	Mechanism & rationale	Example/model	Developmental stage	Key references
“OR/AND” gated CARs	OR-gated	Recognition of either of two antigens to reduce antigen-loss escape	Dual-target glioma CARs	Preclinical; early clinical exploration in GBM	(115)
	AND-gated	Activation only when both antigens are present on the same cell → improved safety	Dual-antigen co-expression studies	Preclinical; strong evidence for reduced off-tumor toxicity	
	IF-THEN gated	First antigen triggers expression of a second CAR → tumor-restricted activation	SynNotch circuits	Advanced preclinical; lead models in GBM	
Armored CARs	Cytokine-secreting CAR T cells	CAR T cells engineered to secrete IL-12, IL-15, IL-18 to enhance persistence, recruit endogenous immunity, and reshape TME	Synthetic-Notch circuits enabling tumor-restricted IL-12 production	Preclinical; early-phase clinical testing with toxicity considerations	(123)
	Dominant-negative/switch receptors	Neutralize inhibitory pathways or convert inhibitory signals (e.g., PD-1–CD28, dnTGFβRII) into stimulatory cues	PD-1–CD28 switch receptor; dnTGFβRII	Preclinical; early-phase trials evaluating safety and feasibility	
Metabolically engineered CARs	Amino-acid metabolism	Enhance amino-acid uptake or endogenous synthesis under depletion	LAT1 overexpression CAR T	Preclinical	(128)
	Glucose metabolism	Improve glucose access; maintain balance between glycolysis and mitochondrial fitness	GLUT1-overexpressing CAR T	Preclinical	
	Lipid metabolism	Prevent lipid-induced dysfunction and ferroptosis	ACAT1-KO CAR T; CD36-KO CAR T	Preclinical	
	Metabolite resistance	Resist immunosuppressive metabolites by degrading enzymes	Lactate; adenosine; kynurenine; ROS resistant CAR T	Preclinical	
Allogeneic CARs	Gene edited healthy-donor T cells	edited to remove TCR/HLA to reduce immunogenicity & improve persistence; scalable & immediately available	TRAC/HLA-edited allogeneic CAR-T products (multiplex CRISPR/TALEN/base-editing)	Early-phase trials	(171)
*In-vivo* engineered CARs	mRNA delivery systems	Direct in-patient delivery of CAR mRNA to T cells → transient CAR expression, repeat dosing, favorable safety	CD8-targeted lipid nanoparticles; fusogenic nanovesicles; pseudoviral fusion particles	Preclinical	(170)
	Viral vectors	in-patient delivery of nanobody- or receptor-targeted viral vectors to T cells → stable CAR integration, durable CAR expression, long-term activity	nanobody-targeted lentiviruses; ESO-T01 CAR T	First-in-human success; early-phase clinical evaluation	
Omics-guided engineered CARs	Genomics	Identify regulators of cytotoxicity, exhaustion, persistence	NR4A-KO CAR T; TOX-KO CAR T	Preclinical	(181)
	Epigenomics	Define exhaustion states and memory-like programs	ATAC-defined T-cell subsets	Preclinical	
	Transcriptomics	Map CAR-T dynamics and TME interactions	scRNA-seq	Preclinical; emerging clinical correlative evidence	
	Proteomics/metabolomic	Identify signaling pathways, metabolic stresses, activation states	Proteomics-guided metabolic engineering	Preclinical	
	Spatial omics	Map the spatial positioning of immune niches, stromal barriers, antigen heterogeneity	Spatial transcriptomic maps of TME	Emerging technology; not yet applied directly to CAR T	

Oncolytic viruses represent a complementary strategy to CAR T-cell therapy in solid tumors. These viruses preferentially replicate within tumor cells, inducing direct oncolysis and promoting local immune activation, thereby reducing immunosuppression within the tumor microenvironment. When combined with CAR T cells, oncolytic viruses can enhance tumor infiltration and effector function. In addition, engineered oncolytic viruses, such as herpes simplex virus-based platforms, can be modified to deliver immunostimulatory payloads or tumor antigens, further improving CAR T-cell targeting and antitumor activity in preclinical models (136).

Cytokine trapping represents another rational combination strategy, as TGF-β-mediated immunosuppression is a major barrier to effective T-cell function within solid tumors. Engineered TGF-β traps prevent ligand-receptor signaling, thereby restoring T-cell proliferation and effector activity. Tumor-localized or CAR T-cell–associated TGF-β trapping has shown improved antitumor responses in preclinical models, offering a promising approach to mitigate TME-driven suppression while minimizing systemic toxicity (137).

## 4 Translational pathway

The next generation of CAR-T therapies for solid tumors will be fundamentally reliant on the identification and validation of robust biomarkers capable of predicting therapeutic response, stratifying patients, and elucidating mechanisms of resistance (138). Insights from hematologic malignancies have demonstrated that treatment outcomes and toxicities can be anticipated through integrated cellular, soluble, and clinical biomarkers; however, this framework has not been fully translated to solid tumor indications due to antigen heterogeneity and the heightened complexity of the tumor microenvironment (TME) (138, 139). Consequently, comprehensive characterization of the solid-tumor TME using multi-omic and spatial profiling approaches is essential to enable rational target selection, biomarker-guided patient selection, and effective clinical translation of CAR-T cell therapies (140).

### 4.1 Biomarkers and biomarker-guided translation in solid tumors

Biomarkers are central to CAR-T clinical translation because they inform patient selection, risk stratification, response prediction, and monitoring for progression/relapse (140). In practice, biomarker needs span the treatment course, including baseline features (e.g., inflammatory cytokines and tumor-burden surrogates such as LDH), markers reflecting CAR-T product phenotype/fitness (differentiation/activation profiles), and indicators linked to toxicity and post-treatment outcomes (138). However, biomarker frameworks from hematologic malignancies are less transferable to solid tumors due to antigen heterogeneity and the added complexity of the tumor microenvironment (TME) (138, 139).

Accordingly, comprehensive characterization of the solid-tumor TME using multi-omic and spatial profiling is increasingly viewed as essential to enable rational target selection, biomarker-guided patient selection, and improved clinical translation (140). Spatial transcriptomics and multiplexed imaging can also delineate how CAR-T cells localize relative to antigen-positive tumor regions and to stromal and myeloid barriers, and spatial patterns of immune-excluded or hypoxic checkpoint-enriched niches may foreshadow therapeutic resistance (40, 41).

### 4.2 Clinical trial design insights

The global number of CAR-T studies has increased steadily, with a noticeable surge starting in 2017. To date, China and the United States lead in the number of clinical trials conducted. In contrast, in the Gulf region, challenges include limited local GMP-compliant manufacturing facilities and prolonged vein-to-vein times (141, 142). Government investment in Saudi Arabia, the UAE, and Qatar creates opportunities to expand regional manufacturing capacity (141). Progressing CAR-T therapies into late-phase trials requires addressing biological and clinical challenges to demonstrate reliable benefit. Improvements in CAR design, manufacturing, and patient selection will be essential for regulatory success and global and regional implementation. Since 2020, there has been a clear shift toward expanding the application of CAR-T therapy to solid tumors. Current trials primarily focus on tumors in the central nervous system, gastrointestinal tract, genitourinary system, and gynecologic cancers (143). Additionally, the repeated evaluation of the same antigen targets across multiple tumor types highlights the rapid and substantial evolution of the field since the introduction of CAR-T cell therapy. While CAR-T cell therapy has shown promise in some solid malignancies, including glioblastoma, the lack of distinct tumor-specific antigens and tumor heterogeneity remains a significant challenge (144, 145). In stromal-rich solid tumors, particularly pancreatic cancer, dense desmoplasia blocks immune-cell entry, and an immunosuppressive microenvironment marked by MHC-I downregulation, regulatory T-cell accumulation, and macrophage-driven inhibition of cytotoxic responses (146, 147). These barriers explain the limited success of current treatments and justify ongoing efforts to develop CAR-T strategies specifically designed to overcome pancreatic cancer’s stromal and immunologic constraints. Most programs remain early-phase, with increasing use of combination and locoregional strategies to balance efficacy and safety (148).

Although CAR-T therapies have achieved regulatory approval for several hematologic malignancies, no CAR-T therapy for solid tumors has yet received marketing authorization. However, ongoing clinical studies have reported encouraging signals of activity in several solid tumor settings, suggesting continued progress toward potential clinical translation (21, 143, 149). Optimizing CAR-T cell constructs to enhance safety and efficacy, for instance, by integrating inducible safety mechanisms such as “suicide switches”, is essential for progression to late-phase clinical trials (150).

Looking ahead, the roadmap for advancing CAR-T cell therapies for solid tumors involves navigating challenges in clinical trial design, regulatory landscapes, and manufacturing logistics. Achieving late-phase trial success and subsequent approval necessitates overcoming hurdles such as poor tumor infiltration, limited CAR-T cell persistence, and the need for manageable toxicity profiles (150). An overview of representative clinical trials evaluating CAR-T cell therapies across major solid tumor indications is provided in **Table 3**.

**Table 3:** Representative clinical trials of CAR-T therapy in solid tumors.

Tumor type	Target antigen	Phase	Outcome	Reference	Notes
Glioblastoma Multiforme (GBM)	HER2	Phase I	Results not yet reported	NCT02442297	Ongoing study
Glioblastoma Multiforme (GBM)	EGFRvIII	Phase I	Completed, no results posted on registry; published report indicates no detectable clinical activity	NCT03726515	Pembrolizumab combination
Glioblastoma Multiforme (GBM)	EGFR806, IL13Rα2	Phase I	ICV delivery feasible and safe; CAR-T cells showed bioactivity with antitumor activity in recurrent GBM.	(199) NCT05168423	
Glioblastoma Multiforme (GBM)	EGFRvIII, EGFR (WT)	Phase I	Rapid radiographic responses after single intraventricular infusion; transient in most patients, with one durable responder.	(200) NCT05660369	CARv3-TEAM-E platform; dual EGFR targeting
Colorectal cancer liver metastases	CEA	Phase I	No severe adverse events observed. In the 6×10^6/kg group, 57% of patients remained relapse-free at 2 years after resection; median follow-up 23 months.	(201) NCT05240950	
Colorectal Carcinoma (CRC)	GUCY2C	Early Phase I	No public results reported to date	NCT04652219	
Pancreatic cancer	Mesothelin	Phase I	Safe and feasible huCART-meso therapy; limited clinical efficacy with only transient stable disease in one patient.	NCT03323944	
Pancreatic cancer	HER2	Phase I	Well tolerated with mainly grade 1–2 toxicities; one partial response (4.5 months) and five cases of stable disease observed; median PFS 4.8 months	(202)	
Gastric or gastroesophageal junction (GEJ) cancer	CLDN18.2	Phase Ib/II	Significantly prolonged progression-free survival with acceptable safety as third-line treatment in advanced gastric/GEJ cancer.	(203) NCT04581473	
Hepatocellular Carcinoma (HCC)	GPC3	Phase I	Acceptable initial safety profile with early signs of antitumor activity observed in advanced HCC	(204)	
Hepatocellular Carcinoma (HCC) and other solid tumors	CD133	Phase I	Acceptable safety profile (≤ grade 3 cytopenias) with antitumor activity observed; 3 partial responses, 14 cases of stable disease, 65.2% disease control rate at 3 months, and median PFS of 5 months	(151)	Multi-tumor cohort
Renal Cell Carcinoma (RCC)	CAIX	Phase I	Significant on-target liver toxicity occurred at low doses, requiring treatment cessation; toxicity was prevented with G250 pretreatment, but no clinical responses were achieved.	(133)	Early CAIX trial; on-target off-tumor toxicity
Metastatic castration-resistant prostate cancer (mCRPC)	PSCA	Phase I	No DLTs at DL1 or DL3; one grade 3 cystitis at DL2. Grade 1–2 CRS in 5/14 patients. Biological activity observed with PSA decline and radiographic responses in 4/14 patients; limited CAR-T persistence.	(205) NCT03873805	PSCA-CAR T; dose-escalation study
Castration-resistant prostate cancer (CRPC)	PSMA	Phase I	All primary safety and feasibility endpoints met. Grade ≥2 CRS occurred in 5/13 patients, including one fatal grade 4 event. PSA declines ≥30% were observed in 4 patients with confirmed CAR T-cell expansion and tumor trafficking.	(206) NCT03089203	
Malignant pleural mesothelioma (MPM)	Mesothelin	Phase I	Intrapleural CAR T-cell infusion was safe and well tolerated, with peripheral persistence >100 days in 39% of patients. With pembrolizumab, median OS was 23.9 months (1-year OS 83%); 8 patients achieved ≥6-month stable disease and 2 had complete metabolic responses.	(207) NCT02414269	Intrapleural delivery; pembrolizumab combination
Non–Small Cell Lung Cancer (NSCLC)	EGFR	Early Phase I	No public results reported to date	NCT05060796	CXCR5-modified CAR T-cells
Small Cell Lung Cancer (SCLC)	DLL3	Phase I	No public results reported to date	NCT05680922	
Small Cell Lung Cancer (SCLC)	α-PD-L1/​DLL3	Phase I	No public results reported to date	NCT06348797	
Malignant Ovarian Cancer	B7H3	Phase I/II	No public results reported to date	NCT05211557	
Platinum-resistant ovarian tumors	B7-H3	Phase I	No public results reported to date	NCT06646627	
Advanced/metastatic gynecologic cancer	CD70	Phase I	No public results reported to date	NCT06215950	
Lung cancer and triple-negative breast cancer	EGFR, B7-H3	Early Phase I	No public results reported to date	NCT05341492	Dual-target CAR-T
Head and Neck Squamous Cell Carcinoma (HNSCC)	CSPG4	Phase I/II	No public results reported to date	NCT06096038	

Clinical trial information compiled from ClinicalTrials.gov and associated primary publications listed in the Reference column.

CRS, cytokine release syndrome; OS, overall survival; PFS, progression-free survival.

Despite the rapid growth of early-phase trials, clinical responses to CAR-T therapy in solid tumors remain limited and often transient. For example, in a phase I study of CD133-targeted CAR-T cells in patients with advanced CD133-positive solid tumors (n = 23), only three patients achieved partial responses (13%), while fourteen experienced stable disease (61%), corresponding to a 3-month disease control rate of 65.2% and a median progression-free survival of 5 months (151), while treatment-related toxicities were primarily reversible hematologic events. These findings illustrate that although measurable antitumor activity can occur, durable tumor regression remains uncommon in most solid-tumor CAR-T trials, highlighting a persistent gap between promising preclinical models and clinical outcomes. These limited outcomes largely reflect the biological barriers outlined earlier, particularly antigen heterogeneity, poor infiltration, and progressive CAR-T dysfunction within the tumor microenvironment.

In addition to limited efficacy, safety considerations remain central to the clinical translation of CAR-T therapy in solid tumors. CAR-T treatment is associated with immune-mediated toxicities such as cytokine release syndrome (CRS) and immune effector cell-associated neurotoxicity syndrome (ICANS), which result from systemic cytokine release and immune activation following CAR-T expansion (152, 153). CRS typically occurs during the first week after infusion and correlates with CAR-T cell proliferation and tumor burden, whereas ICANS manifests as a spectrum of neurological symptoms ranging from mild cognitive disturbances to seizures and cerebral edema (154, 155). In the context of solid tumors, safety concerns are further complicated by on-target, off-tumor toxicity due to shared antigen expression between malignant and healthy tissues, which has been observed in several CAR-T and TCR-based therapies (17, 19, 156).

### 4.3 Manufacturing, scalability, and cost constraints

Beyond biological barriers, several translational bottlenecks limit the progression of CAR-T therapies for solid tumors into late-phase clinical trials. Preclinical models often overestimate efficacy because they do not fully capture the complexity of human solid tumors. In clinical settings, antigen heterogeneity, poor tumor infiltration, and rapid CAR-T cell exhaustion frequently prevent durable tumor control. Together with manufacturing complexity and logistical constraints, these factors contribute to the high attrition rate of solid-tumor CAR-T programs before reaching late-stage development.

CAR-T cell therapy for solid tumors faces substantial manufacturing and logistical barriers that differ markedly from hematologic applications (48). Across early trials, intratumoral and intracerebroventricular routes have shown minimal dose-limiting toxicities even at very high doses (up to 10^10^ cells per infusion), suggesting a wide therapeutic window. However, producing such large cell numbers, particularly when multiple infusions are required, significantly increases the logistical burden and places additional strain on manufacturing capacity (157).

Dose optimization remains a key challenge in solid-tumor CAR-T therapy. Clinical trials use broad and heterogeneous dose ranges, typically 10^5^-10^8^ CAR-T cells/kg, and local administration may permit further escalation (158, 159). Repeated dosing is often necessary, especially in tumors with hostile microenvironments such as CNS cancers, where up to 18 weekly infusions have been reported (157). These multi-dose regimens not only complicate clinical workflow but also require manufacturing facilities to generate large CAR-T batches or maintain cells for sequential infusions, adding substantial production complexity.

Locoregional delivery methods introduce additional logistical considerations. Intratumoral injection, guided by ultrasound or CT, improves local bioavailability and avoids systemic toxicity but requires interventional radiology and may necessitate repeated administrations (160). Intra-arterial delivery, particularly hepatic artery infusion for gastrointestinal malignancies, helps overcome high intratumoral pressure and improves CAR-T persistence but requires procedural expertise and specialized equipment (161).

Beyond delivery logistics, manufacturing itself remains a major barrier. CAR-T therapy relies on leukapheresis, genetic modification, ex vivo expansion, and extensive quality-control testing, making the process slow, labor-intensive, and costly. Solid-tumor patients often have heavily pretreated or dysfunctional T cells, reducing expansion success and prolonging vein-to-vein time. These challenges are amplified for next-generation constructs, such as armored or multi-antigen CAR-Ts, which require larger viral vectors and more complex engineering steps, lowering transduction efficiency and increasing regulatory and quality-control requirements (48, 162).

A key barrier to the widespread adoption of CAR-T cell therapies is their high cost, driven by patient-specific manufacturing, which limits patient access and affordability (138, 163). The development of fully closed and automated manufacturing platforms, while initially expensive, aims to streamline production and reduce labor costs (164). This economic burden is further exacerbated by the protracted production timelines, ranging from two to four weeks for a single dose, which critically impacts patient access and treatment initiation (163).Transitioning toward allogeneic “off-the-shelf” CAR-T cells improves scalability through mass production from healthy donors, reduces costs by eliminating patient-specific manufacturing, enables faster delivery via immediate availability, and simplifies regulatory approval with standardized processes and harmonized guidelines (163–165).

Real-world implementation further highlights the scalability problem. CAR-T therapy requires specialized centers with intensive monitoring capacity, robust supply chain management, and highly trained personnel. In this context, establishing internationally harmonized regulatory guidelines is essential for standardizing the evaluation and approval of these therapies, particularly given the global nature of their development and application (165). Emerging *in vivo* CAR-T engineering platforms, where nanoparticles deliver CAR transgenes directly to T cells within the patient, may eventually help bypass traditional ex vivo production and reduce logistical complexity, but these approaches remain in early development (166).

Overall, manufacturing and logistical constraints, including dose production limits, the need for specialized delivery routes, repeated infusion schedules, and complex ex vivo engineering, represent major obstacles to scaling CAR-T therapy for solid tumors. Addressing these challenges will be essential to enable broader clinical translation and integration of next-generation CAR-T technologies.

### 4.4 Delivery routes and patient selection

Solid tumors provide distinct challenges, such as a highly immunosuppressive microenvironment, considerable antigen heterogeneity, and physical barriers that obstruct CAR-T cell penetration and efficacy, hence diminishing the effectiveness of numerous hematologic biomarkers (149).

Furthermore, the physical barriers presented by the blood-brain barrier and the dense extracellular matrix within glioblastoma tumors impede CAR-T cell trafficking and uniform distribution throughout the tumor mass (167). These anatomical constraints necessitate intratumoral or intracerebroventricular delivery strategies and restrict eligibility to patients amenable to neurosurgical access, illustrating how the delivery route directly governs patient selection in CNS malignancies.

Despite promising preclinical results, clinical responses to CAR-T therapy in solid tumors remain inconsistent, underscoring the importance of optimizing delivery strategies and carefully selecting patients most likely to benefit from route-specific CAR-T approaches (138, 150).

### 4.5 Regulatory, economic, and regional determinants of CAR-T translation

Importantly, regulatory and implementation challenges do not occur in isolation but are closely linked to the biological and translational barriers described earlier, including antigen heterogeneity, complex manufacturing requirements, and tumor microenvironment constraints. Beyond biological and engineering constraints, the global translation of CAR-T therapies for solid tumors is shaped by regional regulatory frameworks, economic capacity, and manufacturing infrastructure. These implementation challenges are closely intertwined with scientific barriers such as complex CAR designs, manufacturing scalability, and the need for advanced engineering strategies to overcome the hostile tumor microenvironment. Asia now accounts for a substantial proportion of global CAR-T clinical trials, largely driven by China, and plays a prominent role in early-phase CAR-T development, including solid tumor indications. This shift is supported by more flexible regulatory pathways that facilitate faster trial initiation. However, translation beyond early-phase development remains challenging (168).

The use of CAR-T cell therapy worldwide is limited by high manufacturing costs and the need for specialized clinical infrastructure, which reduces affordability and access, especially in lower-income regions where treatment costs can far exceed national GDP per capita. In Asia, several local strategies, such as on-site CAR-T manufacturing, patient assistance programs, pharmaceutical subsidies, and support from charities and donors, have been introduced to help address these challenges. Despite these efforts, hidden costs related to facilities, staffing, and quality control still make it difficult to scale these therapies and ensure fair access (169). These factors illustrate how regulatory and economic frameworks must evolve alongside scientific advances in CAR-T engineering and manufacturing in order to enable effective clinical translation.

Despite this leadership, China, followed by the United States, accounts for the largest share of global CAR-T clinical studies; however, most trials worldwide, including those conducted in Asia, remain in early development stages. Advancement to late-phase trials is limited by complex manufacturing processes, very high per-patient costs often reaching several hundred thousand US dollars, and high attrition rates. Moreover, CAR-T development also faces funding challenges and uncertain efficacy, particularly for smaller companies, with academic institutions supporting more than half of the registered trials in China. By contrast, stricter regulatory requirements for solid-tumor CAR-T therapies in the United States and Europe have slowed late-phase translation, despite rapid growth in early-phase solid-tumor CAR-T studies (143).

## 5 Future directions

While CAR T-cell therapy has transformed the treatment of hematologic malignancies, its extension to solid tumors will require fundamental advances in cell engineering, manufacturing, and systems-level design. Emerging strategies aim to overcome current translational barriers by enabling *in vivo* CAR-T generation, developing scalable allogeneic platforms, and leveraging multi-omic, spatial, and computational frameworks to guide rational CAR design. In parallel, addressing regulatory, infrastructural, and access-related challenges will be essential to ensure the safe, equitable, and globally scalable implementation of next-generation CAR-T therapies for solid tumors.

### 5.1 *In vivo* CAR-T engineering

*In vivo* CAR-T engineering offers an alternative strategy by delivering CAR-encoding nucleic acids directly into T cells within the patient, eliminating the need for ex vivo manipulation. Two vector classes dominate this approach. mRNA delivery systems, including CD8-targeted lipid nanoparticles, fusogenic nanovesicles, and pseudoviral fusion particles, enable rapid, transient CAR expression with a favorable safety profile and allow repeat dosing, making them attractive for solid tumors. In contrast, viral vectors, such as nanobody-targeted lentiviruses, achieve stable integration and long-term CAR expression (170, 171). Several of these platforms are now advancing toward clinical evaluation.

The ESO-T01 trial represents the early clinical demonstration of *in vivo* CAR-T generation in humans. In this platform, a targeted lentiviral vector delivers a BCMA-specific CAR construct directly to circulating T cells within the patient, enabling *in situ* generation of CAR-T cells without leukapheresis or ex vivo manufacturing. Early clinical observations reported in the initial study support feasibility, including detectable CAR expression and *in vivo* expansion, with preliminary antitumor activity and manageable toxicity in relapsed or refractory multiple myeloma. ESO-T01 is being further evaluated in early-phase clinical studies (ClinicalTrials.gov identifiers NCT06691685 and NCT06791681) (172). Collectively, these findings position *in vivo* CAR programming as a potential route to shorten vein-to-vein time and improve scalability by bypassing individualized manufacturing.

### 5.2 Allogeneic CAR-T platforms

Autologous CAR-T cells, generated through leukapheresis, ex vivo viral transduction, and expansion, remain the current standard but are limited by high cost, long manufacturing times, and variable product quality in heavily pretreated patients (173). Allogeneic (“off-the-shelf”) CAR-T cells use healthy-donor T cells that are pre-manufactured, cryopreserved, and available immediately. Their major challenges include graft-versus-host disease (GVHD), host-versus-graft rejection, and risks related to gene editing and scalability. Newer platforms employ multiplex genome editing, including CRISPR, TALENs, base editing, and prime editing, to disrupt endogenous TCR and HLA molecules, reduce immunogenicity, and improve persistence (174). Several allogeneic CAR-T products have been evaluated in solid tumors, with early trials reporting manageable safety profiles and preliminary antitumor activity (171).

### 5.3 Multi-omics and spatial profiling to guide CAR-T design

Multi-omics technologies provide a multidimensional view of CAR T-cell biology, revealing how these cells function, adapt, and fail within solid tumors (175). Genomic and CRISPR-based screens identify genes that regulate CAR T-cell cytotoxicity, exhaustion, and persistence, guiding engineering strategies such as NR4A- or TOX-knockout approaches (176).

Epigenomic profiling (ATAC-seq, single-cell chromatin mapping) defines exhaustion-associated epigenetic states and helps identify memory-like subsets associated with durable responses (177).

Transcriptomic analyses, particularly scRNA-seq, map dynamic transcriptional programs during therapy and uncover interaction networks between CAR T cells and suppressive tumor or stromal cells (178). Proteomic and metabolomic studies reveal signaling pathways, metabolic stresses, and activation states that inform strategies such as metabolic reprogramming or armoring (179, 180). Integrating datasets (e.g., RNA-seq + CITE-seq + ATAC-seq) enables tracking of CAR T-cell evolution over time and improves discovery of features linked to long-term persistence or treatment failure, although further computational development is needed to combine multi-omics at scale (181).

Spatial omics adds a necessary layer by revealing where specific immune, stromal, and tumor populations reside within intact tumor tissue (182). By mapping the spatial positioning of immunosuppressive niches, stromal barriers, and regions of differential antigen expression, spatial transcriptomics can identify microanatomical barriers that prevent CAR-T-cell infiltration or induce exhaustion. Although spatial profiling has not yet been widely applied to CAR-T-cell therapy, early work in immune-checkpoint inhibitor studies suggests that spatially resolved data will be critical for designing next-generation CAR-T cells capable of navigating the physical and immunologic architecture of solid tumors (181).

Single-cell and bulk multi-omic datasets have substantially advanced our comprehension of the profound diversity and dynamic nature of the tumor microenvironment. Recent reviews in single-cell multi-omics underscore how integrating transcriptomic, epigenomic, proteomic, and metabolomic profiles at single-cell resolution unveils intricate heterogeneity across malignant, immune, and stromal compartments that elude detection in bulk analyses (39, 183, 184). These investigations demonstrate that multi-omic approaches enable the simultaneous profiling of cellular identity, activation status, lineage trajectories, and regulatory networks, thereby facilitating the association of distinct cellular states with therapeutic resistance and immune evasion (38, 39, 185).

In this context, pan-cancer single-cell atlases further refine immune and stromal lineage resolution, identifying heterogeneous cancer-associated fibroblast and T-cell states associated with immunotherapy response (186–188). Together, multi-omics and spatial technologies provide a rational framework for engineering more precise and resilient CAR T-cell therapies, linking molecular states to functional performance and enabling the design of CARs optimized for persistence, selectivity, and activity within the complex solid tumor microenvironment (181).

### 5.4 Computational modeling and artificial intelligence in CAR-T design

Computational modelling and artificial intelligence (AI) are increasingly used to analyze multi-omic and clinical datasets to guide CAR-T cell therapy design, including antigen selection, prediction of therapeutic responses, and evaluation of potential off-tumor toxicities (189, 190). Computational models have been used to examine how antigen heterogeneity influences CAR-T therapy outcomes in solid tumors and to estimate antigen-expression thresholds required for effective tumor control (191, 192).

Building on these computational analyses, machine learning (ML) approaches have been applied to single-cell and spatial transcriptomic datasets to identify potential antigen combinations for CAR-T targeting. Kwon et al. leveraged single-cell transcriptomic data and ML algorithms to pinpoint optimal multi-antigen combinations for logic-gated CAR-T constructs, thereby enabling engineered CAR-T cells to sustain antitumor efficacy despite nonuniform expression of individual antigens (193). Meanwhile, Kuang et al. employed ML on spatial and single-cell transcriptomic data to reconstruct spatial antigen maps, thereby delineating heterogeneous tumor niches that guide CAR-T design (194).

Taken together, these studies highlight how AI-assisted analysis of multi-omic datasets can inform data-driven CAR-T design strategies aimed at addressing antigen heterogeneity in solid tumors (191, 192, 194).

### 5.5 Regulatory and implementation outlook

**Figure 2:**
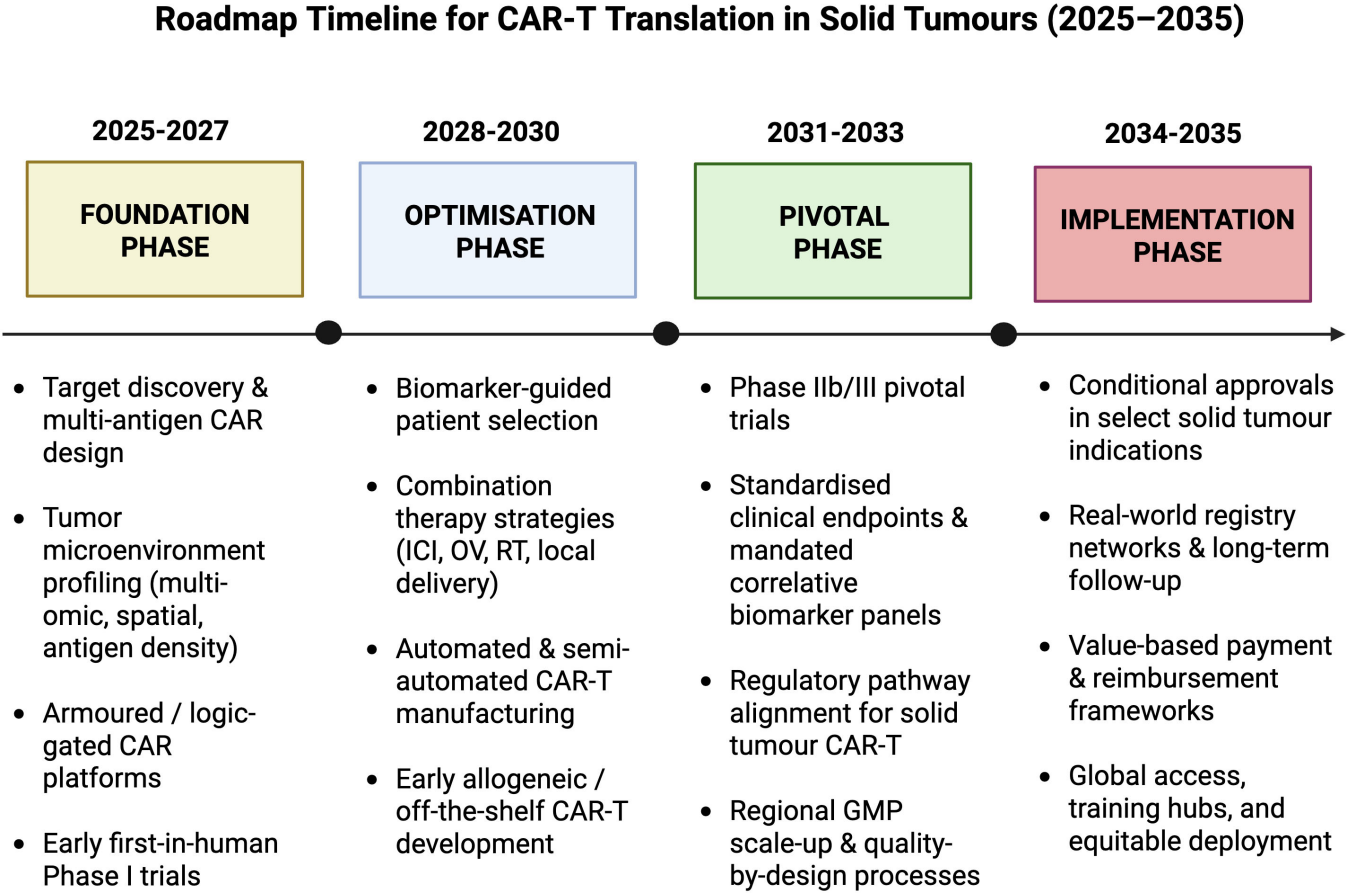
Roadmap timeline for translation of CAR-T in solid tumors (2025-2035). This figure illustrates the anticipated translational progression of CAR-T therapies for solid tumors, outlining key developmental stages from foundational target and engineering advances through optimization and pivotal clinical evaluation to regulatory integration and clinical implementation. Created with BioRender.com.

Implementation of CAR-T therapy across Gulf countries is constrained by high costs, limited infrastructure, and the absence of uniform reimbursement frameworks (138, 195). Advanced cell-therapy centers are concentrated in major urban hubs, often necessitating domestic or international travel for patients. These disparities raise ethical concerns regarding equitable access and underscore the need for decentralized, point-of-care manufacturing and standardized automated platforms to reduce logistical complexity and cost (196, 197). To address these challenges, investment in decentralized manufacturing and point-of-care facilities could reduce transportation times and costs, thereby improving patient access and streamlining therapy delivery. Furthermore, the adoption of standardized automated platforms within decentralized manufacturing models holds promise for optimizing workflows, enhancing process robustness, and promoting scalability, thereby improving the efficacy and cost-effectiveness of CAR-T cell products **Figure 2** (164). Improving access to CAR-T therapy requires reducing logistical and financial barriers while expanding decentralized production models. Ensuring equitable availability is essential for ethical and global implementation.

## 6 Conclusions

A key contribution of this review is a systems-level conceptual roadmap that explicitly connects dominant mechanisms of failure in solid tumors to matched engineering strategies and biomarker/computational approaches that can accelerate translation. Chimeric antigen receptor (CAR) T-cell therapy is a rapidly evolving immunotherapeutic approach for the treatment of solid tumors, and the field has made substantial progress over the past decade. Despite the growing number of clinical trials, long-term durable responses remain limited by biological challenges, including antigen heterogeneity, poor tumor penetration, and the immunosuppressive tumor microenvironment. Importantly, the limited progression of CAR-T therapies for solid tumors into late-phase clinical trials reflects persistent translational barriers rather than a lack of biological rationale. Many early-phase studies demonstrate feasibility and acceptable safety profiles but fail to achieve durable tumor control due to insufficient CAR-T persistence, heterogeneous antigen expression, and the suppressive tumor microenvironment. Addressing these barriers will be critical for translating early clinical signals into durable therapeutic benefit. Major engineering advances, such as *in vivo* CAR programming, logic-gated designs, and armored constructs, are rapidly expanding clinical feasibility and are expected to significantly enhance tumor infiltration, persistence, and resistance to immunosuppression by 2030. Achieving this potential will require sustained investment in tumor-specific antigen discovery, interdisciplinary collaboration across immunology, bioengineering, artificial intelligence, and translational oncology, and the development of scalable manufacturing and regulatory frameworks. Collectively, the integration of immunotherapy with digital and computational technologies represents a critical translational imperative to enable equitable, safe, and globally scalable CAR T-cell therapies.
